# Canonical NF-κB p65, but Not p105, Contributes to IL-1β-Induced IL-8 Expression in Cardiac Fibroblasts

**DOI:** 10.3389/fimmu.2022.863309

**Published:** 2022-04-20

**Authors:** Masashi Mizuno, Rei Nakano, Saki Nose, Moeka Matsumura, Yasuyuki Nii, Kentaro Kurogochi, Hiroshi Sugiya, Masami Uechi

**Affiliations:** ^1^Japan Animal Specialty Medical Institute, Tsuzuki, Yokohama, Japan; ^2^Laboratory for Mucosal Immunity, Center for Integrative Medical Sciences, RIKEN Yokohama Institute, Yokohama, Japan; ^3^Laboratory of Veterinary Radiotherapy, Department of Veterinary Medicine, College of Bioresource Sciences, Nihon University, Fujisawa, Japan

**Keywords:** cardiac fibroblasts, inflammation, interleukin 1β, interleukin 8, NF-κB p65

## Abstract

Cardiac fibroblasts participate in the inflammatory process of heart diseases as sentinel cells of the cardiac tissue. In this study, we investigated the effect of the proinflammatory cytokine, interleukin 1β (IL-1β), on the expression of interleukin 8 (IL-8), which contributes to the induction of innate immunity *via* the activation and recruitment of innate immune cells, such as neutrophils, to the site of inflammation in canine cardiac fibroblasts. IL-1β mediates IL-8 mRNA expression and protein release in a dose- and time-dependent manner. The IL-β-mediated IL-8 protein release and mRNA expression were inhibited by 2-[(aminocarbonyl)amino]-5-(4-fluorophenyl)-3-thiophenecarboxamide, an inhibitor of the transcription factor, nuclear factor (NF)-κB. In cells treated with IL-1β, NF-κB p65 and p105 were transiently phosphorylated, indicating the activation of NF-κB. However, IL-1β failed to induce IL-8 mRNA expression in the cells transfected with p65 small interfering RNA (siRNA), but not in those transfected with p105 siRNA. These observations suggest that IL-1β induces IL-8 expression *via* the activation of NF-κB p65 in canine cardiac fibroblasts.

## Introduction

The myocardium consists of several cell types, namely, cardiomyocytes, cardiac fibroblasts, endothelial cells, and smooth muscles. Of these, cardiac fibroblasts have a large cell population (1), although the relative number of cardiomyocytes and non-cardiomyocytes likely varies among different species (2). Cardiac fibroblasts are involved in many aspects of cardiac function, such as maintaining normal cardiac structure, cell signaling, and electro-mechanical function of the heart (3–5). They also participate in the inflammatory process of heart diseases as sentinel cells of the cardiac tissue (6, 7). They respond to pathogen-associated molecular patterns (PAMPs) or danger-associated molecular patterns (DAMPs) that secrete a wide variety of proinflammatory cytokines and chemokines (6).

Interleukin-8 (IL-8), also known as the C-X-C motif chemokine ligand 8 (CXCL8), is a proinflammatory ELR^+^ C-X-C chemokine. IL-8 plays an important role in the induction of innate immunity *via* the activation and recruitment of innate immune cells, such as neutrophils, to the site of inflammation (8, 9). IL-8 is also involved in angiogenesis by promoting the proliferation, growth, and viability of vascular endothelial cells (10, 11). IL-8 is produced in and secreted from various cell types, namely, blood monocytes, alveolar macrophages, and non-immune cells, such as endothelial cells and fibroblasts (12–16). IL-8 expression is stimulated by a variety of stimuli, namely, pro-inflammatory cytokines, although it is virtually undetectable in unstimulated cells (17). The pro-inflammatory cytokine interleukin-1β (IL-1β), which contributes to acute or chronic inflammation by mediating the expression and secretion of cytokines and chemokines in inflamed tissues (18, 19), has been shown to mediate the expression and secretion of IL-8 as demonstrated in various cells, namely, fibroblasts (14–16, 20, 21).

Nuclear factor kappa B (NF-κB) is a family of transcription factors critical for regulating a variety of cellular functions, namely, the immune response and inflammation (22–24). NF-κB consists of five subunits: p65 (RelA), RelB, c-Rel (Rel), p50/p105 (NF-κB1), and p52/p100 (NF-κB2). These subunits form homo- and heterodimer complexes, resulting in up to 15 dimers with varying cellular functions (22–24). NF-κB activity is mainly regulated by its interaction with inhibitory proteins, such as IκB. In its latent form, dimers of NF-κB occur as a complex with IκB in the cytoplasm, which is an inactive form. NF-κB is activated by the disruption of the interaction between IκB and NF-κB. The dimeric complex of NF-κB released by the degradation of IκB translocates into the nucleus and induces the expression of immune and inflammatory genes by binding to their promoters (22–24). The NF-κB pathway consists of two pathways: canonical and noncanonical. Pro-inflammatory cytokines, such as IL-1β and tumor necrosis factor α (TNF-α), activate the canonical NF-κB signaling pathway, while other stimuli, such as the CD40 ligand, activate the non-canonical pathway (22–24).

In this study, we demonstrated that IL-1β mediated the expression of IL-8 *via* the activation of NF-κB p65 in canine cardiac fibroblasts.

## Materials and Methods

### Materials

Dulbecco’s modified Eagle’s medium with 1 g/L glucose (DMEM-LG), 4-(2-hydroxyethyl)-1-piperazineethanesulfonic acid (HEPES), phenylmethanesulfonyl fluoride (PMSF), sodium fluoride, and fetal bovine serum (FBS) was purchased from FUJIFILM Wako Chemical Corp. (Osaka, Japan). Recombinant canine IL-1β and 2-[(aminocarbonyl)amino]-5-(4-fluorophenyl)-3-thiophenecarboxamide (TPCA-1) were purchased from Kingfisher Biotech, Inc. (Saint Paul, MN) and MedChemExpress (Monmouth Junction, NJ, USA), respectively. Lipofectamine 2000 and TRIzol were purchased from Life Technologies (Carlsbad, CA, USA). CELLBANKER 1 plus medium, Thermal Cycler Dice Real Time System II, TP900 Dice Real Time v4.02B, SYBR Premix Ex Taq II, and PrimeScript RT Master Mix were purchased from TaKaRa Bio, Inc. (Shiga, Japan). Rabbit monoclonal anti-phosphorylated p65 (93H1), anti-total p65 (D14E12), anti-phosphorylated p105 (18E6), anti-total p105 (D4P4D), and mouse monoclonal anti-total IκBα (L35A5) antibodies were purchased from Cell Signaling Technology Japan, K.K. (Tokyo, Japan). Anti-β-actin mouse monoclonal antibody (AC74), small interfering RNA (siRNA) for p65 and p105, and scrambled siRNA were obtained from Sigma-Aldrich Inc. (St. Louis, MO, USA). Horseradish peroxidase (HRP)-conjugated anti-rabbit and anti-mouse IgG antibodies, the ECL western blotting Analysis System, and Amersham ImageQuant 800 were purchased from Cytiva Japan (Tokyo, Japan). Mini-PROTEAN TGX gel, polyvinylidene difluoride (PVDF) membranes, and iCycler were obtained from Bio-Rad (Hercules, CA). The complete mini ethylenediaminetetraacetic acid (EDTA)-free protease inhibitor mixture and Block Ace were purchased from Roche (Mannheim, Germany) and KAC Co., Ltd. (Hyogo, Japan), respectively. An enzyme-linked immunosorbent assay (ELISA) kit for canine IL-8 (#CA8000), a freezing vessel (BICELL), and StatMate IV were purchased from R&D Systems, Inc. (Minneapolis, MN), Nihon Freezer Co., Ltd. (Tokyo, Japan), and ATMS (Tokyo, Japan), respectively.

### Cell Culture

Canine primary cardiac fibroblasts were purchased from Cell Biologics (Chicago, IL, USA). The thawed-out cell suspension was transferred into a centrifuge tube contained DMEM-LG containing 20% FBS. After centrifugation at 300×*g* for 1 min and removal of the supernatant, the cell pellet was resuspended in DMEM-LG containing 20% FBS and transferred into a 75-cm^2^ culture flask. The cells were maintained in static culture in an incubator at 5% carbon dioxide (CO_2_) and 37°C using DMEM-LG supplemented with 20% fetal calf serum, and the medium was changed once a week. When the fibroblasts reached 90–95% confluence, they were harvested using 0.25% trypsin-EDTA. The collected cardiac fibroblasts were suspended using CELLBANKER 1 plus medium at a density of 2 × 10^6^ cells/500 μl, divided into 500 μl each, and placed into a sterilized serum tube. The tubes were then placed into the freezing vessel BICELL and cryopreserved at −80°C. Before being used in the experiments, serum tubes were removed from the BICELL vessel and immersed in a water bath at 37°C. The thawed-out cell suspension was transferred into a centrifuge tube contained DMEM-LG containing 20% FBS. After centrifugation at 300×*g* for 1 min and removal of the supernatant, the pellet was suspended in DMEM-LG containing 20% FBS and transferred into a 75-cm^2^ culture flask. Static cultures were then performed under the same conditions as those before cryopreservation.

### Real-Time Quantitative Reverse Transcription-Polymerase Chain Reaction (qRT-PCR)

Real-time RT-PCR was performed as previously reported (25–36). Total RNA was extracted from cultured canine cardiac fibroblasts using TRIzol reagent, following the instructions of the manufacturer. Synthesis of first-strand cDNA was performed with 500 ng of total RNA using PrimeScript RT Master Mix. Real-time RT-PCR was performed with 2 μl of first-strand cDNA in 25 μl (total reaction volume), SYBR Premix Ex Taq II, and primers targeting canine IL-8 or hypoxanthine phosphoribosyltransferase 1 (HPRT1), the housekeeping gene (**Table 1**). Real-time RT-PCRs of “no-template” controls or “no-reverse transcription” controls were performed with 2 μl of RNase- and DNA-free water or 2 μl of each RNA sample, respectively. PCR was conducted using the Thermal Cycler Dice Real-Time System II. The protocol was as follows: one cycle of denaturation at 95°C for 30 s, 40 cycles of denaturation at 95°C for 5 s, and annealing/extension at 60°C for 30 s. The results were analyzed by the second derivative maximum method and the comparative cycle threshold (ΔΔCt) method using real-time RT-PCR analysis software. The amplification of HPRT1 from the same amount of cDNA was used as an endogenous control, whereas cDNA amplification from canine cardiac fibroblasts at time 0 was used as the calibration standard.

**Table 1:** Primer sequences for quantitative reverse transcription-polymerase chain reaction (qRT-PCR).

Gene Name	Gene bank ID	Primer sequences
IL-8	NM_001003200.1	F: 5′-CACCTCAAGAACATCCAGAGCT-3′
		R: 5′-CAAGCAGAACTGAACTACCATCG-3′
HPRT1	NM_001003357.2	F: 5’-GGAGCATAATCCAAAGATGGTCAA-3’
		R: 5’-TCAGGTTTATAGCCAACACTTCGAG-3’

### Western Blotting

Western blotting was performed as described previously (25–27, 29–36). Canine cardiac fibroblasts were lysed in 20 mM HEPES buffer (pH 7.4) containing 1 mM PMSF, 10 mM sodium fluoride, and a complete mini EDTA-free protease inhibitor cocktail. Protein concentrations were determined using the Bradford method (37) and adjusted. After boiling at 98°C for 5 min in the sodium dodecyl sulfate buffer, the extracted protein samples were loaded into separate lanes of 12 or 7.5% Mini-PROTEAN TGX gel and electrophoretically separated. Separated proteins were transferred to PVDF membranes, treated with Block Ace for 50 min at room temperature, and incubated with the following primary antibodies [phosphorylated p-65 (p-p65, 1:1,000), total p65 (t-p65, 1:1,000), phosphorylated p-105 (p-p105, 1:1,000), total p105 (t-p105, 1:1,000), total IκBα (t-IκBα, 1:1,000), and β-actin (1:10,000)] for 120 min at room temperature. After washing, membranes were incubated with HRP-conjugated anti-rabbit or anti-mouse IgG (1:10,000) for 90 min at room temperature. Immunoreactivity was detected using the ECL western blotting Analysis System, and chemiluminescent signals of the membranes were measured using an Amersham ImageQuant 8000.

### ELISA for IL-8

Canine cardiac fibroblasts were seeded at a density of 3.0 × 10^5^ cells/well in 6-well culture plates. After starvation for 24 h, fibroblasts were treated with IL-1β for 0–24 h and the culture medium was collected. The IL-8 concentration in the culture medium was assayed using an ELISA kit, according to the instructions of the manufacturer.

### Transfection of siRNA

siRNA transfection was performed as previously described with slight modifications (25–27, 29–36). Canine cardiac fibroblasts were seeded at a density of 1 × 10^5^ cells/35 mm dish or 5 × 10^5^ cells/90 mm dish, and transfected using Opti-MEM containing 3 μl/ml Lipofectamine 2000 and 2 μM p65, p105, or scramble siRNA (**Table 2**) for 24 h. After transfection, the medium was changed to DMEM-LG containing 20% FBS and the cultures were maintained in an incubator with 5% CO_2_ at 37°C for 2 d. The efficiency of siRNA-mediated knockdown was validated by western blotting.

**Table 2:** Sequences for the transfection of small interfering RNA (siRNA).

Gene Name	Gene bank ID	siRNA sequences
p65	XM_014121307.2	GCAUCUCCCUGGUCACCAA
p105	AB183419.1	CUGCAAAGGUUAUUGUUCA

### Statistical Analysis

All statistical analyses were performed using EZR (38), a graphical user interface for R (R Foundation for Statistical Computing, Vienna, Austria). More precisely, it is a modified version of the R commander designed to add statistical functions frequently used in biostatistics. Data from all experiments are shown as the mean ± standard error and were analyzed using one-way analysis of variance (ANOVA) with Holm–Bonferroni multiple comparisons test as a *post hoc* test. Statistical significance was set at p <0.05.

## Results

### IL-1β-Mediated IL-8 mRNA Expression and Protein Release

**Figure 1:**
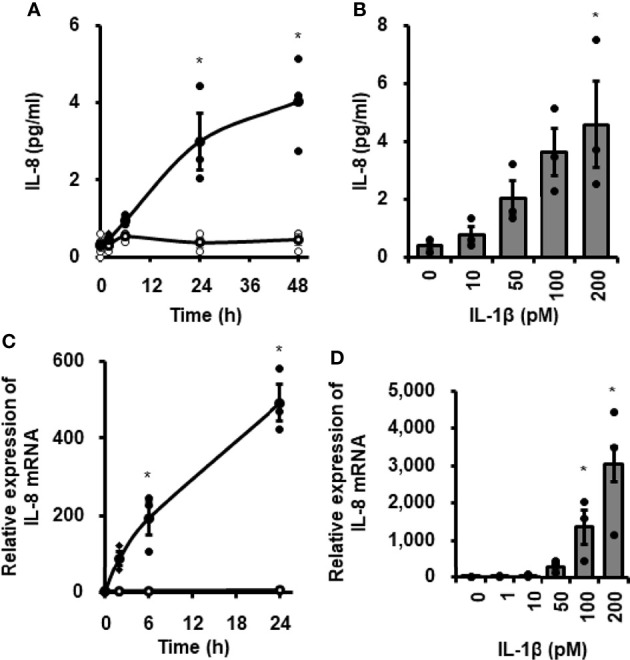
IL-1β-mediated IL-8 release and IL-8 mRNA expression in canine cardiac fibroblasts. Time-dependent increase in IL-8 protein release **(A)** and IL-8 mRNA expression levels **(C)** in fibroblasts treated with (closed circle) or without (open circle) canine recombinant IL-1β (100 pM). Dose-dependent IL-8 protein release **(B)** and IL-8 mRNA expression levels **(D)** in fibroblasts treated with the indicated concentrations of IL-1β for 24h. HRPT1 was used as an internal standard and the expression levels of IL-8 mRNA in IL-1β-stimulated fibroblasts were compared with the expression at 0h. Results have been represented as mean ± standard error (SE) from biological triplicates. *P <0.05.

When canine cardiac fibroblasts were incubated with 100 pM IL-1β for 0–48 h, the concentration of IL-8 in the incubation medium increased in a time-dependent manner (**Figure 1A**). In fibroblasts treated with various concentrations of IL-1β (0–200 pM) for 24 h, IL-1β stimulated IL-8 release in a dose-dependent manner (**Figure 1B**). We examined the effect of IL-1β on IL-8 mRNA expression in canine cardiac fibroblasts. As shown in **Figures 1C, D**, IL-1β stimulated IL-8 mRNA expression in a time- and dose-dependent manner. These observations suggest that IL-1β induces IL-8 expression and release in canine cardiac fibroblasts.

### Inhibitory Effect of TPCA-1 on IL-1β-Mediated IL-8 mRNA Expression and Protein Release

**Figure 2:**
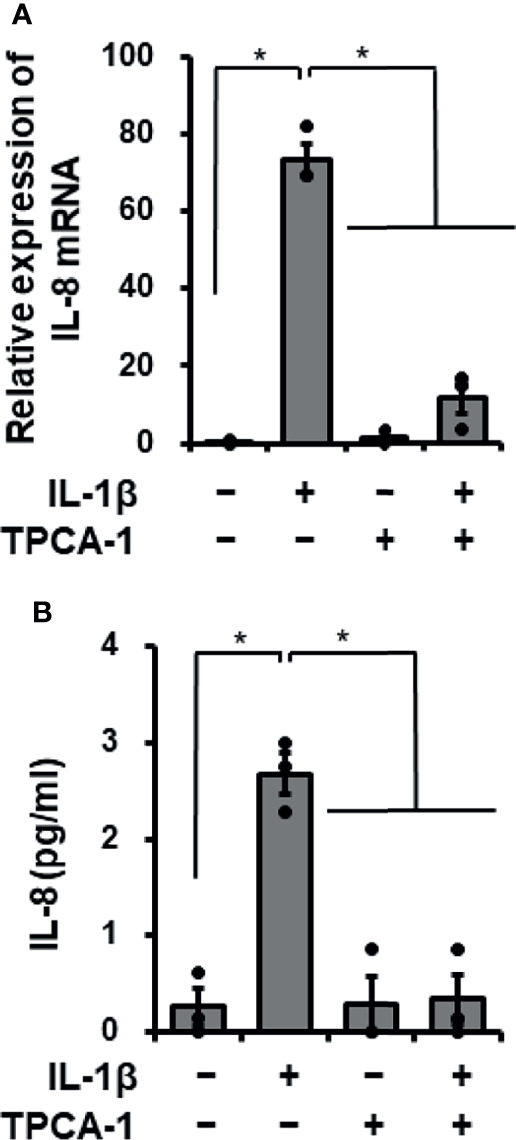
Effect of the NF-κB inhibitor TPCA-1 on IL-1β-mediated IL-8 mRNA expression. Canine cardiac fibroblasts were pretreated with or without TPCA-1 (10 µM) for 1 h and subsequently stimulated with or without IL-1β (100 pM) for 24 h. After stimulation, IL-8 mRNA expression levels **(A)** and the release of IL-8 **(B)** were determined. HRPT1 was used as an internal standard and the expression levels of IL-8 mRNA in IL-1β-stimulated fibroblasts were compared with the expression at 0 h. Results have been represented as mean ± standard error (SE) from biological triplicates. *P <0.05.

IL-1β is known to mediate the mRNA expression of various proteins *via* the activation of the transcription factor NF-κB. We then examined the involvement of NF-κB in IL-1β-mediated IL-8 expression in canine cardiac fibroblasts using the NF-κB inhibitor TPCA-1. In fibroblasts pretreated with TPCA-1 (10 μM) for 1 h, the effects of IL-1β on IL-8 mRNA expression (**Figure 2A**) and IL-8 release (**Figure 2B**) were clearly attenuated. These observations suggest that NF-κB activation is involved in the IL-1β-induced IL-8 mRNA expression in canine cardiac fibroblasts.

### IL-1β-Mediated Activation of NF-κB

**Figure 3:**
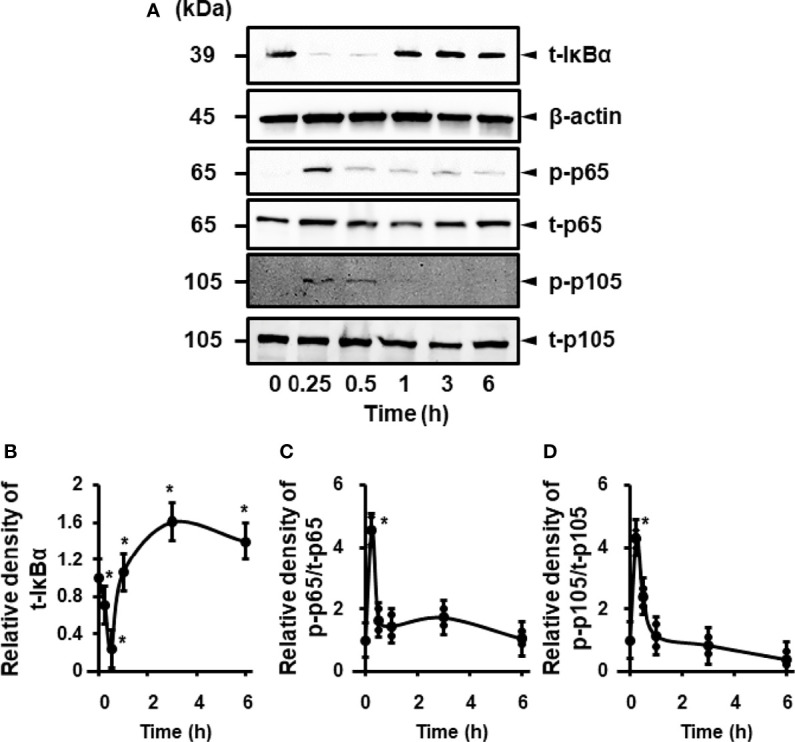
IL-1β-mediated activation of NF-κB. **(A)** Representative western blotting results of levels of total IκBα (t-IκBα), phosphorylated p65 (p-p65), total p65 (t-p65), phosphorylated p105 (p-p105), and total p105 (t-p105) in canine cardiac fibroblasts treated with IL-1β (100 pM). β-actin was used as an internal standard. Time-dependent changes of levels of t-IκBα, p-p65 and p-p105 were observed. Relative levels of t-IκBα **(B)**, [p-p65]/[t-p65] **(C)**, and [p-p105]/[t-p105] **(D)** in IL-1β-stimulated fibroblasts compared to the levels at 0h. Results have been represented as mean ± SE from biological triplicates. *P <0.05.

**Figure 4:**
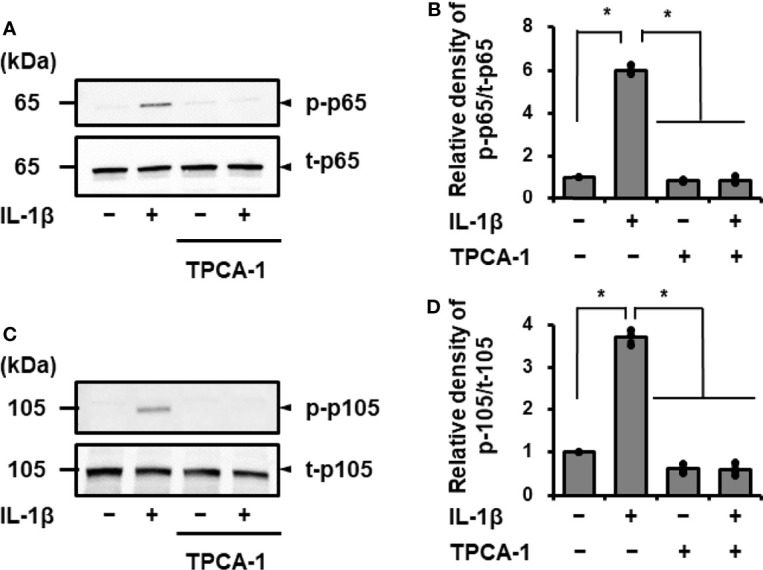
Effect of the NF-κB inhibitor TPCA-1 on IL-1β mediated phosphorylation of p65 and p105. Canine cardiac fibroblasts were pretreated with or without TPCA-1 (10 µM) for 1 h and stimulated with IL-1β (100 pM) for 15 min. Representative western blotting results of inhibitory effect of TPCA-1 on IL-1β-mediated phosphorylation of p65 **(A)** and p105 **(C)**, and relative levels of [p-65]/[t-65] **(B)** and [p-105]/[t-105] **(D)** as compared to those without the inhibitor and IL-1β. Results have been represented as mean ± SE from biological triplicates. *P <0.05.

Next, we examined the effects of IL-1β on the activation of NF-κB in canine cardiac fibroblasts. When fibroblasts were stimulated with 100 pM IL-1β for 0–300 min, IκBα degradation was observed at 15–30 min (**Figures 3A, B**). Phosphorylation of p65 and p105 was also observed 15–30 min after stimulation, indicating the activation of NF-κB by IL-1β (**Figures 3A, C, D**). In fibroblasts pretreated with the NF-κB inhibitor, TPCA-1, for 1 h, IL-1β-mediated phosphorylation of p65 and p105 was clearly attenuated (**Figure 4**). These observations suggest that IL-1β induces IL-8 expression *via* the activation of NF-κB.

### IL-1β-Mediated IL-8 mRNA Expression in Cells Transfected With siRNA of p65 or p105

**Figure 5:**
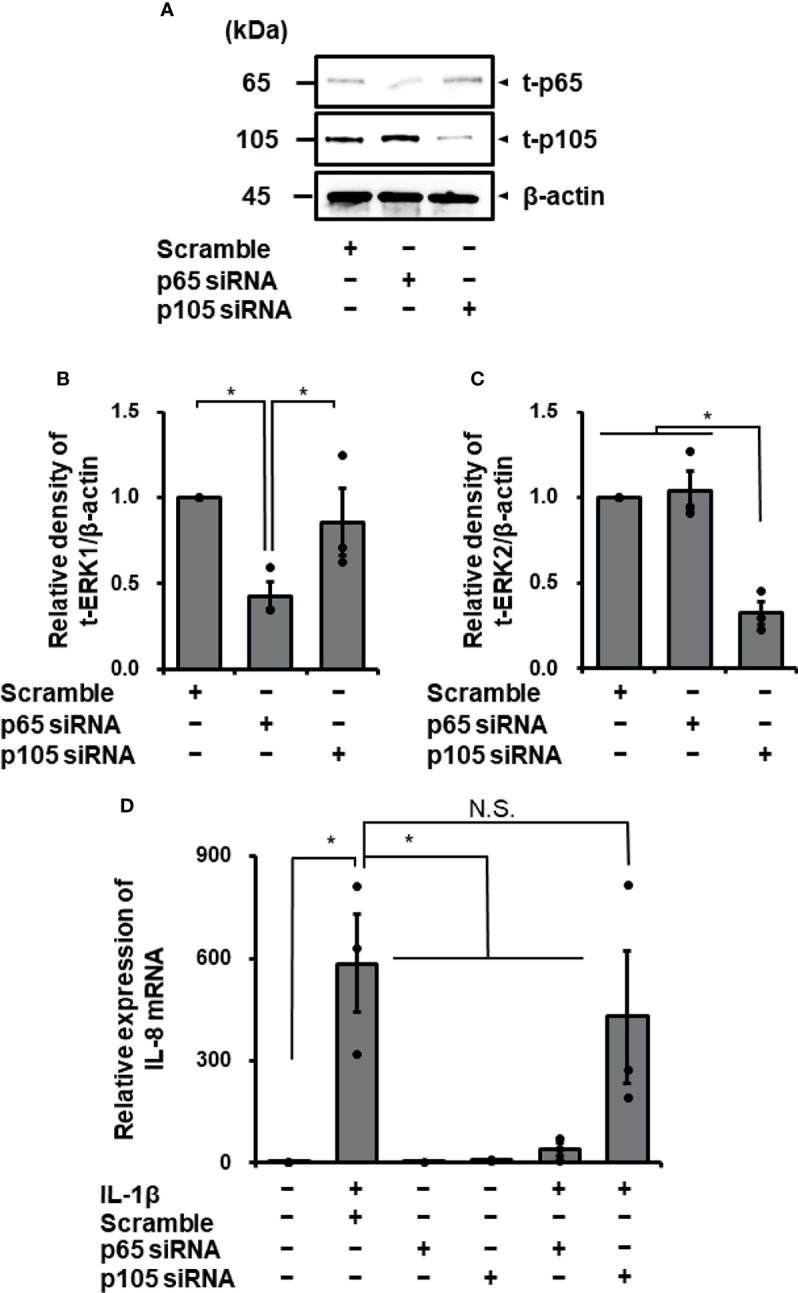
Transfection with p65 siRNA attenuated IL-1β-mediated IL-8 mRNA expression in canine cardiac fibroblasts but not that with p105 siRNA. In canine cardiac fibroblasts transfected with p65, p105, and scrambled siRNAs, expression of t-p65, t-105, and β-actin was detected by western blotting. The expression of p65 or p105 was reduced in fibroblasts transfected with p65 or p105 siRNA, respectively, but not in fibroblasts transfected with scrambled siRNA. β-actin was used as an internal standard. Representative results **(A)** and relative density of protein expression of t-p65 **(B)** or t-p105 **(C)** in siRNA-transfected fibroblasts compared with those in scrambled siRNA-transfected fibroblasts are depicted. **(D)** Cardiac fibroblasts transfected with p65, p105 and scrambled siRNAs were incubated with or without IL-1β (100 pM) for 24 h After the incubation, IL-8 mRNA expression was determined. HRPT1 was used as an internal standard. Transfection with p65 siRNA resulted in reduction of IL-1β-mediated IL-8 mRNA expression, while p105 and scrambled siRNA-transfection did not. Results have been represented as mean ± standard error (SE) from biological triplicates. *P <0.05; N.S, Not Significant.

To confirm the contribution of NF-κB to IL-1β-induced IL-8 expression, we examined the effect of IL-1β on IL-8 mRNA expression in p65 or p105 knockdown fibroblasts using siRNA transfection. In fibroblasts transfected with p65 or p105 siRNA, p65 or p105 protein expression was clearly reduced compared with that in control fibroblasts transfected with scramble RNA (**Figures 5A–C**). IL-1β-mediated IL-8 mRNA expression was clearly decreased in fibroblasts transfected with p65 siRNA compared to that in the control (**Figure 5D**). However, p105 siRNA transfection failed to attenuate the IL-1β-mediated IL-8 mRNA expression (**Figure 5D**). Taken together, it is likely that NF-κB p65 activation contributes to IL-1β-mediated expression of IL-8, but not p105, in canine cardiac fibroblasts.

## Discussion

In this study, we demonstrated that IL-1β mediates IL-8 production in canine cardiac fibroblasts. IL-8 is a critical inflammatory mediator that functions as a chemotactic factor for neutrophils and lymphocytes, and an angiogenic factor (39–41). In human patients with myocardial infarction, which is associated with an intense inflammatory response that ultimately leads to healing and scar formation, a significant increase in serum IL-8 levels has been reported (42–44). An increase in serum IL-8 concentrations has also been reported in human patients undergoing cardiac transplantation (45, 46). Ischemia followed by reperfusion (I/R) induces a complex series of inflammatory reactions, resulting in myocardial injury in animals (47). In a rabbit and rat model of myocardial I/R injury, an increase in serum IL-8 levels was observed (48, 49). In a dog treated with I/R, IL-8 mRNA expression in the myocardium was enhanced (50). These observations suggested that IL-8 is involved in cardiac inflammation and myocardial injury. Recombinant canine IL-8 markedly increases the adhesion of neutrophils to isolated canine cardiac myocytes *in vitro*, but the effect of IL-8 is blocked by antibodies directed against IL-8 (50). In a rabbit model of I/R injury, neutralization of IL-8 using a monoclonal antibody against IL-8 resulted in a reduction in the degree of necrosis (51). In a model of I/R injury, treatment with pterostilbene, a candidate anti-inflammatory substance, attenuated serum IL-8 levels, which was associated with a reduction in infarct size and myocardial apoptosis (49). Therefore, it is likely that IL-8 mediates inflammation *via* I/R injury.

IL-1β is a pro-inflammatory mediator in acute and chronic inflammation, which provokes the synthesis and expression of a variety of secondary inflammatory mediators (52, 53). In experimental mouse models, IL-1β was reported to be highly expressed in cardiac tissues following acute myocardial infarction (54).

The blockade of IL-1 signaling attenuates heart failure after acute myocardial infarction in both mice and humans (55, 56). These observations suggest that IL-1β plays an important role in MI pathogenesis in myocardial infarction. IL-1β is considered important for recruiting leukocytes, especially neutrophils and monocytes, to the infarcted area after myocardial infarction (54, 55). Therefore, IL-1β-mediated IL-8 expression and release in cardiac fibroblasts may contribute to inflammation.

In this study, we demonstrated that NF-κB p65 contributes to IL-1β-mediated IL-8 expression in canine cardiac fibroblasts. In the absence of stimuli, p65 exists in the cytoplasm in a heterodimeric form with the p50 subunit or a homodimeric form in the inactivated state bound to an inhibitory IκB protein, such as IκBα, inhibiting translocation of NF-κB into the nucleus (57–60). In cells stimulated with cytokines, including IL-1β, the IκB protein is degraded by the proteasome, and both dimeric forms of NF-κB are translocated into the nucleus to induce the expression of immune and inflammatory genes by binding to their promoters (23, 47, 61). We demonstrated that IκBα was degraded in cells stimulated with IL-1β. Degradation of IκB is regulated by IκB kinases (23, 47, 61). In our study, IL-1β-mediated IL-8 mRNA expression and protein release in cells treated with the IκB kinase inhibitor TPCA-1 (62). Therefore, it is likely that IκB kinase activation is an important upstream of the activation of p65 in canine cardiac fibroblasts. In our study, IL-1β stimulated the phosphorylation of p105. p105 is a precursor protein that generates the p50. Phosphorylation of p105 is involved in signal-induced p105 processing to p50 (63–67). However, IL-1β-mediated IL-8 mRNA expression occurred in the p105-knockdown cells. Therefore, it is unlikely that p50/p105 is involved in the IL-1β-mediated IL-8 mRNA expression. NF-κB p65 homodimers preferentially bind to the promoter regions of the IL-8 gene (59, 68). Thus, it is conceivable that the p65 homodimer functions as a regulator of IL-8 expression in canine cardiac fibroblasts stimulated with IL-1β. In this study, it is still unclear how IL-8 gene and its transcription are regulated in a genomic context in IL-1β-cardiac fibroblasts. Elucidation of genome-wide regulation in this pathway is indeed our next research subject.

In conclusion, IL-1β mediates IL-8 expression in canine cardiac fibroblasts, and activation of NF-κB p65 contributes to this IL-1β-mediated IL-8 expression. These results support the idea that cardiac fibroblasts are involved in the inflammatory processes of heart diseases as sentinel cells of the cardiac tissue (6, 7).

## Data Availability Statement

The original contributions presented in the study are included in the article/**Supplementary Material**. Further inquiries can be directed to the corresponding author.

## Author Contributions

MasM, R.N., H.S., and M.U. designed and planned the experiments. MasM, R.N., S.N., MoeM, N.Y. and K.K. prepared, measured, and analyzed the samples. MasM, R.N., H.S., and M.U. interpreted the data and wrote the paper. All authors listed have made a substantial, direct, and intellectual contribution to the work and approved it for publication.

## Funding

This work was supported in part by a Grant-in-Aid for Scientific Research (grant no. 18K14594, RN) from the Ministry of Education, Science, Sports, and Culture of Japan (https://www.jsps.go.jp/j-grantsinaid/). The funders had no role in the study design, data collection and analysis, decision to publish, or preparation of the manuscript.

## Conflict of Interest

The authors declare that the research was conducted in the absence of any commercial or financial relationships that could be construed as a potential conflict of interest.

## Publisher’s Note

All claims expressed in this article are solely those of the authors and do not necessarily represent those of their affiliated organizations, or those of the publisher, the editors and the reviewers. Any product that may be evaluated in this article, or claim that may be made by its manufacturer, is not guaranteed or endorsed by the publisher.
