# Supplementary material for: Canonical NF-κB p65, but Not p105, Contributes to IL-1β-Induced IL-8 Expression in Cardiac Fibroblasts
Source: Front Immunol. 2022 Apr 20;13:863309. doi: 10.3389/fimmu.2022.863309 (PMC9065446; doi:10.3389/fimmu.2022.863309)
Supplement: Supplementary file 1 [file DataSheet_1.pdf]

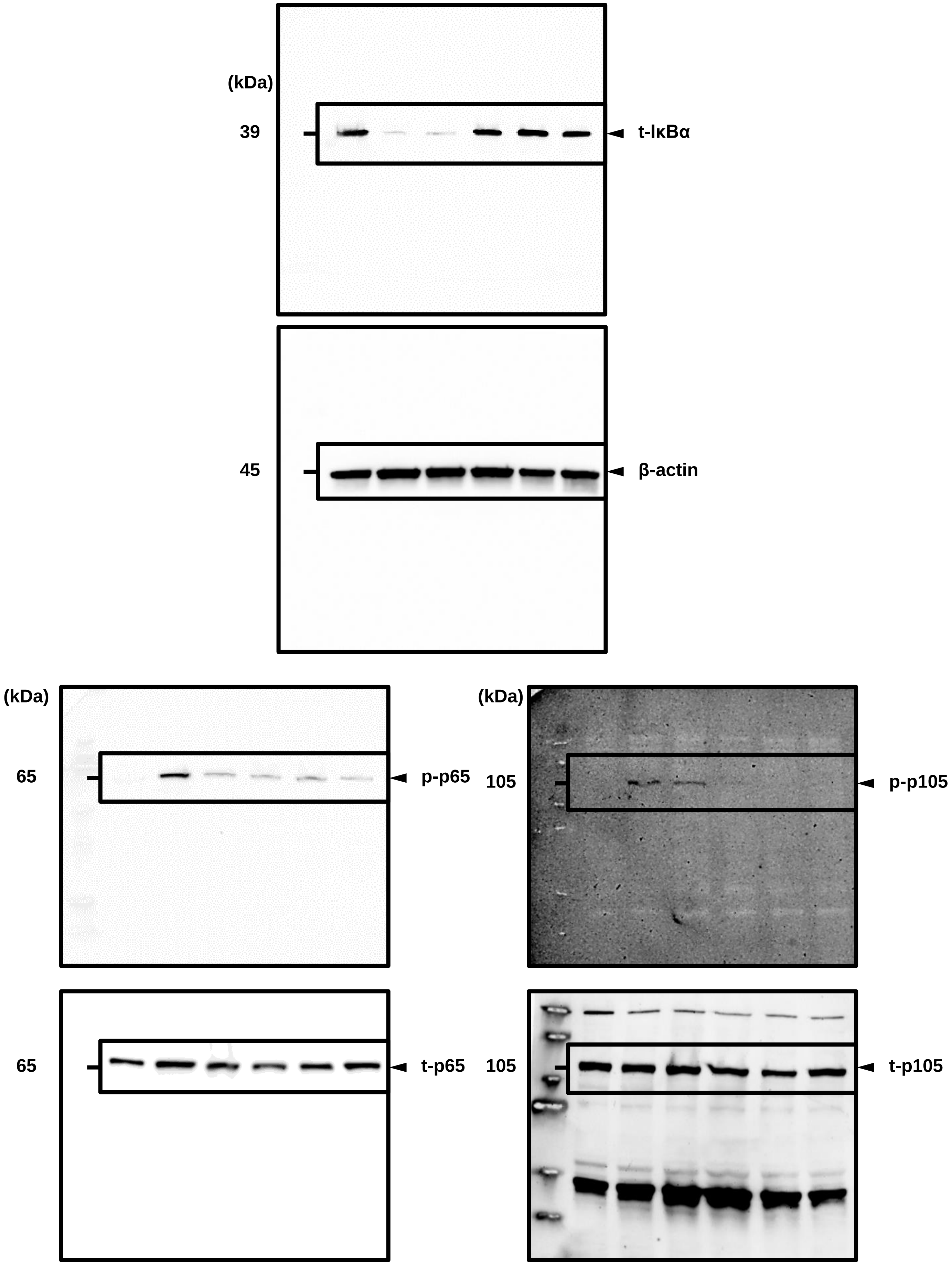

Uncropped images for the blots shown in Fig. 3a

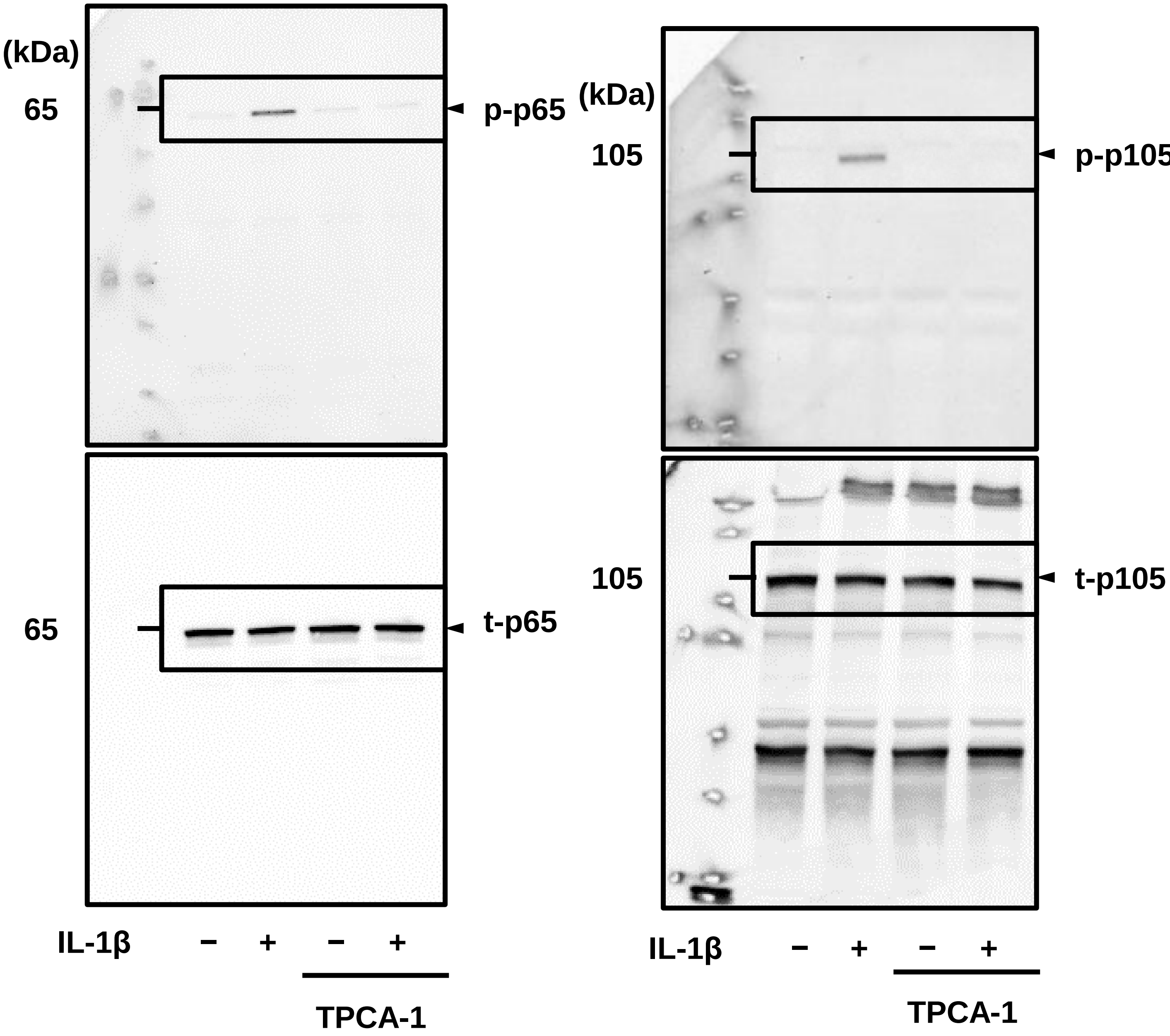

Uncropped images for the blots shown in Fig. 4a and c

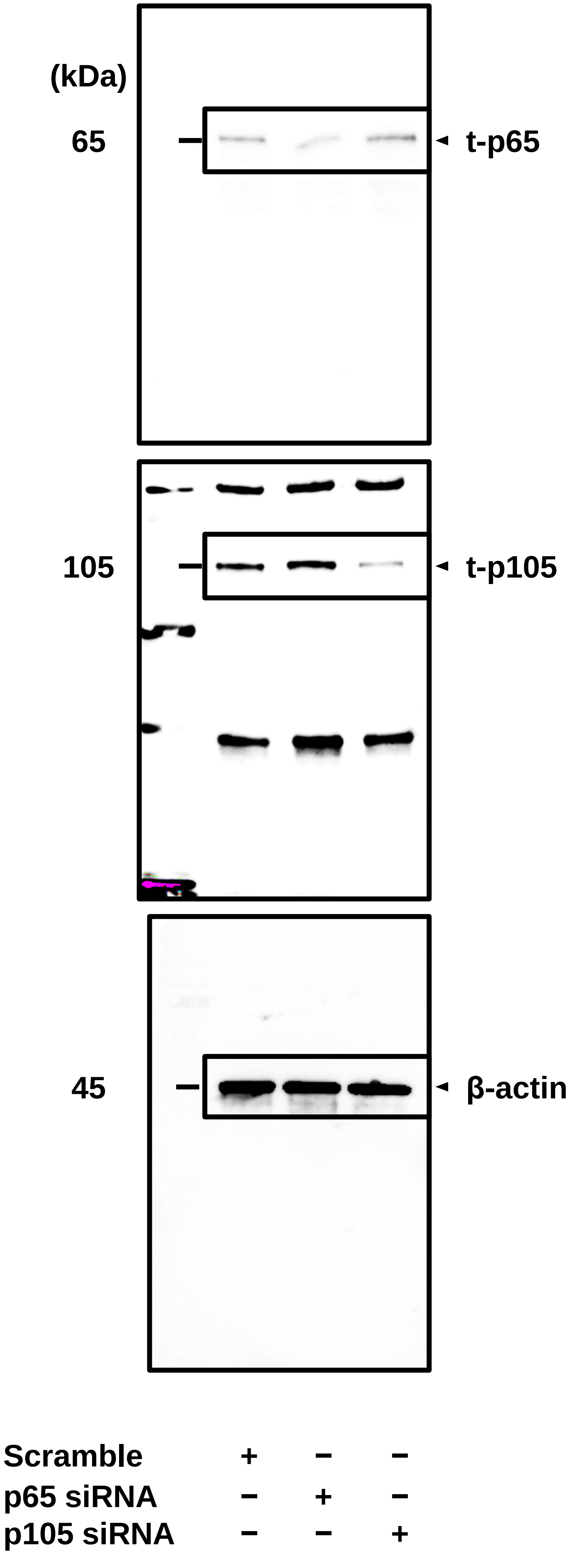

Uncropped images for the blots shown in Fig. 5a
